# Effect of various acupuncture courses for upper limb motor dysfunction after ischemic stroke: a Bayesian network meta-analysis

**DOI:** 10.3389/fnagi.2025.1668293

**Published:** 2025-11-17

**Authors:** Can Wang, Pei Yu, Yuqi Tang, Yaning Liu, Jiangwei Shi, Xuhui Yang, Zihan Yin, Ling Zhao

**Affiliations:** 1 Acupuncture and Tuina School, Chengdu University of Traditional Chinese Medicine, Chengdu, Sichuan, China; 2 First Teaching Hospital of Tianjin University of Traditional Chinese Medicine, Tianjin, China

**Keywords:** ischemic stroke, upper limb motor dysfunction, acupuncture, course, network meta-analysis

## Abstract

**Background:**

Acupuncture has been widely used in the treatment of post-ischemic stroke upper limb motor dysfunction (PIS-ULMD). However, previous studies have reported substantial variability in acupuncture courses, and the lack of a clearly defined optimal course has impeded further improvement in therapeutic outcomes. Studies show that treatment course is a key factor in acupuncture’s dose-effect relationship. The Specification of Formulation and Evaluation for the Clinical Practice Guideline of Acupuncture and Moxibustion [CAAM-2019(001)], issued by the China Association of Acupuncture-Moxibustion (CAAM), points out that current domestic acupuncture clinical practice guidelines lack evidence-based temporal parameters, resulting in clinicians’ reliance on personal experience and inconsistent treatment outcomes. Herein, we conducted network meta-analysis to compare the effectiveness of diverse acupuncture courses for PIS-ULMD treatment.

**Methods:**

Ten databases were searched from their inception to March 21, 2025. Randomized controlled trials (RCTs) on acupuncture for PIS-ULMD were screened. The Cochrane Collaboration Risk of Bias (RoB 2) tool was used to assess the risk of bias in the included studies. The primary outcome was the change in the Fugl-Meyer Assessment-Upper Extremity (FMA-UE) scale before and after treatment. All meta-analysis was performed using RevMan 5.3, STATA (V14.0) and Aggregate Data Drug Information System (ADDIS) (V1.16.6). The Grading of Recommendations Assessment, Development and Evaluation (GRADE) system was applied to evaluate the quality of evidence for each outcome measure.

**Results:**

A total of 67 RCTs involving 5,635 PIS-ULMD patients were included. The pairwise meta-analysis indicated that acupuncture combined with conventional therapy resulted in higher FMA-UE scores compared to conventional therapy alone (*n* = 5,635; MD = 6.95, 95% CI: 5.89–8.00). Network meta-analysis results recommended that 8-week acupuncture course is the most effective acupuncture course. However, the evidence quality was low to critically low.

**Conclusion:**

Acupuncture combined with conventional therapy significantly improves upper limb motor function in PIS-ULMD patients. For enhancing upper limb motor function, an 8-week acupuncture regimen may be more appropriate, particularly for patients in the subacute phase and severe PIS-ULMD. However, the overall evidence quality was low, it is recommended additional well-designed RCTs with larger sample sizes to validate these findings.

**Systematic review registration:**

https://www.crd.york.ac.uk/PROSPERO/, Identifier CRD420251022808.

## 1 Introduction

Stroke is an acute neurological deficit caused by cerebrovascular circulatory disorders, ranking as the third leading cause of death and disability worldwide. The latest Global Burden of Disease (GBD) report on stroke reveals a significant rise in both stroke-related deaths and persistent disability cases over the past 15 years, alongside an increasing incidence trend among the under-55 population (GBD 2021 Stroke Risk Factor Collaborators, 2024), ischemic stroke constitutes the most prevalent pathological subtype, accounting for 85% of all stroke cases (Pluta et al., 2021). Upper limb motor impairment represents one of the most prevalent sequelae following ischemic stroke, significantly compromising patients’ ability to perform activities of daily living. This neurological deficit typically manifests as a constellation of motor control deficits, coordination disorders, sensory disturbances, and impaired manual dexterity. Current research indicates that approximately 80% of acute stroke patients develop upper limb motor dysfunction (Rodgers et al., 2019). The upper limbs present greater rehabilitation challenges due to their responsibility for finer motor control and more extensive representation in the sensorimotor cortex (Li et al., 2023). Evidence shows that 50–60% of patients still exhibit persistent upper limb motor dysfunction at 6 months (Wade et al., 1983). Patients with PIS-ULMD demonstrated significantly divergent rehabilitation outcomes based on impairment severity. In cases of mild motor impairment, approximately 79% achieved complete functional recovery (Nakayama et al., 1994). For moderate-to-severe cases, only 3.6% achieved complete recovery (Koh et al., 2015). This condition significantly impairs daily functioning and substantially diminishes patients’ quality of life.

Acupuncture demonstrates unique therapeutic advantages in managing post-ischemic stroke hemiplegia. The World Health Organization (WHO) recommends acupuncture as a complementary and alternative strategy for both ischemic stroke treatment and post-stroke care improvement. Clinical trials and meta-analyses have demonstrated that acupuncture exhibits significant therapeutic effects in enhancing balance function, reducing spasticity, increasing muscle strength, and improving overall health status following ischemic stroke (Chavez et al., 2017). According to the 2023 Chinese Guidelines for the Diagnosis and Treatment of Acute Ischemic Stroke, acupuncture may be considered as a therapeutic option based on individual patient circumstances and preferences (Class I recommendation, Level B evidence) (Chinese Society of Neurology, Chinese Stroke Society, 2024). Contemporary research has revealed that acupuncture intervention can enhance regional cerebral circulation and nutrient metabolism, improve tissue perfusion, and accelerate the repair processes of neuronal tissues in stroke patients (Zhu et al., 2024). Acupuncture has demonstrated clinically confirmed efficacy in the treatment of PIS-ULMD.

The manifestation of acupuncture effects requires sufficient time and cumulative stimulation dosage. A treatment course that is too short may fail to achieve the expected therapeutic outcomes, while an excessively prolonged course may not yield additional benefits and could even lead to acupuncture tolerance (Cheng et al., 2022). Studies have revealed that neurological functional recovery after ischemic stroke is most pronounced within the first 3 months, particularly in post-stroke motor recovery, and generally does not extend beyond 6 months (Yang et al., 2016). This finding holds significant clinical implications for the rehabilitation of PIS-ULMD patients, suggesting that the efficacy of rehabilitation interventions for focal ischemic brain injury diminishes over time (Kwakkel et al., 2006). Therefore, achieving optimal temporal alignment between the treatment course and the enhanced phase of neural remodeling is crucial for promoting clinical neurological recovery. However, current published clinical studies on acupuncture intervention for PIS-ULMD exhibit considerable variability in treatment duration, ranging from several weeks to months (Wang et al., 2024). Moreover, researchers typically lack standardized reference criteria for selecting acupuncture treatment courses, and the application of different durations may substantially influence subsequent changes in upper limb motor function. The optimal acupuncture intervention duration remains to be further elucidated.

Network meta-analysis (NMA) enables simultaneous analysis of both indirect and direct evidence to rank different acupuncture treatment courses and ultimately identify the optimal regimen for managing PIS-ULMD. This study employs pairwise meta-analysis to evaluate and compare various acupuncture treatment courses for PIS-ULMD, with subsequent ranking of their therapeutic efficacy to determine the optimal treatment protocol, thereby providing more effective clinical management strategies.

## 2 Materials and methods

The study design followed the PRISMA-NMA guideline (Supplementary file S1) and has been registered with PROSPERO (Registration No. CRD420251022808).

### 2.1 Inclusion criteria and exclusion criteria

#### 2.1.1 Types of studies

All published randomized controlled trials (RCTs) reported in English or Chinese were included without regional or publication restrictions. Non-randomized clinical studies, quasi-randomized controlled trials, cluster randomized trials, case reports, and studies without extractable data were excluded.

#### 2.1.2 Types of participants

Patients diagnosed with PIS-ULMD, regardless of gender, aged 18 years or older were included. Patients with upper limb motor dysfunction caused by non-ischemic stroke etiologies were excluded.

#### 2.1.3 Types of interventions

The literatures that adopted acupuncture as the main intervention measure were included. All acupuncture modalities (manual acupuncture, electroacupuncture, warm needling, fire needling, scalp acupuncture, etc.) were eligible regardless of needling techniques or acupoint selections. Interventions involving acupoint catgut embedding, acupoint injection, bee venom acupuncture, bloodletting therapy, cupping, or herbal medicine were excluded.

#### 2.1.4 Types of control group

Conventional treatments included pharmacotherapy or rehabilitation therapy, as well as sham acupuncture.

#### 2.1.5 Types of outcome measures

We included studies reporting one or more of the following prespecified outcomes. Our systematic review primarily aimed to compare and rank the efficacy and safety of different acupuncture treatment courses for PIS-ULMD. Accordingly, the primary outcome measure was the change in the Fugl-Meyer Assessment for Upper Extremity (FMA-UE) scale before and after treatment, with secondary outcomes including changes in the National Institutes of Health Stroke Scale (NIHSS) and the Modified Barthel Index (MBI) scores before and after treatment.

### 2.2 Search strategy

To ensure comprehensive literature retrieval, this study systematically searched eight databases, including PubMed, Web of Science, Embase, the Cochrane Library, ClinicalTrials.gov, China National Knowledge Infrastructure (CNKI), VIP Database, Wanfang Data, the Chinese Biomedical Literature Database (CBM), and the Chinese Clinical Trial Registry. The search period spanned from each database’s inception to March 21, 2025. To ensure an effective search, a combination of Medical Subject Headings (MeSH) and free words will be used. Boolean operators “AND” and “OR” were strategically combined between search terms. The search strategy was adapted to each database’s specific requirements, with PubMed serving as the representative example. Detailed search strategies are provided in Table 1.

**Table 1:** PubMed search strategy.

Steps	Search
#1	(“Stroke”[Mesh]) OR ((((((((((((((((((((((((((((Strokes[Title/Abstract]) OR (Cerebrovascular Accident[Title/Abstract])) OR (Cerebrovascular Accidents[Title/Abstract])) OR (Cerebral Stroke[Title/Abstract])) OR (Cerebral Strokes[Title/Abstract])) OR (Stroke, Cerebral[Title/Abstract])) OR (Strokes, Cerebral[Title/Abstract])) OR (Cerebrovascular Apoplexy[Title/Abstract])) OR (Apoplexy, Cerebrovascular[Title/Abstract])) OR (Vascular Accident, Brain[Title/Abstract])) OR (Brain Vascular Accident[Title/Abstract])) OR (Brain Vascular Accidents[Title/Abstract])) OR (Vascular Accidents, Brain[Title/Abstract])) OR (Cerebrovascular Stroke[Title/Abstract])) OR (Cerebrovascular Strokes[Title/Abstract])) OR (Stroke, Cerebrovascular[Title/Abstract])) OR (Strokes, Cerebrovascular[Title/Abstract])) OR (Apoplexy[Title/Abstract])) OR (CVA (Cerebrovascular Accident[Title/Abstract]))) OR (CVAs (Cerebrovascular Accident[Title/Abstract]))) OR (Stroke, Acute[Title/Abstract])) OR (Acute Stroke[Title/Abstract])) OR (Acute Strokes[Title/Abstract])) OR (Strokes, Acute[Title/Abstract])) OR (Cerebrovascular Accident, Acute[Title/Abstract])) OR (Acute Cerebrovascular Accident[Title/Abstract])) OR (Acute Cerebrovascular Accidents[Title/Abstract])) OR (Cerebrovascular Accidents, Acute[Title/Abstract]))
#2	(((“Acupuncture”[Mesh]) OR (“Acupuncture Therapy”[Mesh])) OR (“Acupuncture, Ear”[Mesh])) OR (((((((((((((((((((Pharmacopuncture[Title/Abstract]) OR (Acupuncture Treatment[Title/Abstract])) OR (Acupuncture Treatments[Title/Abstract])) OR (Treatment, Acupuncture[Title/Abstract])) OR (Therapy, Acupuncture[Title/Abstract])) OR (Pharmacoacupuncture Treatment[Title/Abstract])) OR (Treatment, Pharmacoacupuncture[Title/Abstract])) OR (Pharmacoacupuncture Therapy[Title/Abstract])) OR (Therapy, Pharmacoacupuncture[Title/Abstract])) OR (Acupotomy[Title/Abstract])) OR (Acupotomies[Title/Abstract])) OR (warm acupuncture[Title/Abstract])) OR (warm needle[Title/Abstract])) OR (needle warming[Title/Abstract])) OR (dry needle[Title/Abstract])) OR (abdominal needle[Title/Abstract])) OR (scalp needle[Title/Abstract])) OR (Electroacupuncture[Title/Abstract])) OR (needle[Title/Abstract]))
#3	(((((“Dyskinesias”[Mesh]) OR (“Hemiplegia”[Mesh])) OR (“Spasm”[Mesh])) OR (“Movement Disorders”[Mesh])) OR (“Paralysis”[Mesh])) OR ((((((((((((((((((((((((((((Dyskinesia[Title/Abstract]) OR (Abnormal Movements[Title/Abstract])) OR (Abnormal Movement[Title/Abstract])) OR (Movement, Abnormal[Title/Abstract])) OR (Movements, Abnormal[Title/Abstract])) OR (Hemiplegias[Title/Abstract])) OR (Monoplegia[Title/Abstract])) OR (Monoplegias[Title/Abstract])) OR (Spasms[Title/Abstract])) OR (Muscle Spasm[Title/Abstract])) OR (Muscle Spasms[Title/Abstract])) OR (Spasm, Muscle[Title/Abstract])) OR (Spasms, Muscle[Title/Abstract])) OR (Muscular Spasm[Title/Abstract])) OR (Muscular Spasms[Title/Abstract])) OR (Spasm, Muscular[Title/Abstract])) OR (Spasms, Muscular[Title/Abstract])) OR (Movement disorders[Title/Abstract])) OR (Movement Disorder[Title/Abstract])) OR (Dyskinesia Syndromes[Title/Abstract])) OR (Dyskinesia Syndrome[Title/Abstract])) OR (Movement Disorder Syndromes[Title/Abstract])) OR (Movement Disorder Syndrome[Title/Abstract])) OR (Paralyses[Title/Abstract])) OR (Palsy[Title/Abstract])) OR (Palsies[Title/Abstract])) OR (Plegia[Title/Abstract])) OR (Plegias[Title/Abstract]))
#4	(“Upper Extremity”[Mesh]) OR (((((((((((((Extremities, Upper[Title/Abstract]) OR (Upper Extremities[Title/Abstract])) OR (Extremity, Upper[Title/Abstract])) OR (Membrum superius[Title/Abstract])) OR (Upper Limb[Title/Abstract])) OR (Limbs, Upper[Title/Abstract])) OR (Limb, Upper[Title/Abstract])) OR (Upper Limbs[Title/Abstract])) OR (shoulder[Title/Abstract])) OR (elbow[Title/Abstract])) OR (wrist[Title/Abstract])) OR (hand[Title/Abstract])) OR (finger[Title/Abstract]))
#5	#3 OR #4
#6	(“Randomized Controlled Trials as Topic”[Mesh]) OR (((((((((((((Clinical Trials, Randomized[Title/Abstract]) OR (Trials, Randomized Clinical[Title/Abstract])) OR (Controlled Clinical Trials, Randomized[Title/Abstract])) OR (Randomized Controlled Trial[Title/Abstract])) OR (randomized clinical trials[Title/Abstract])) OR (randomized controlled clinical trial[Title/Abstract])) OR (RCT[Title/Abstract])) OR (controlled clinical trial[Title/Abstract])) OR (randomized[Title/Abstract])) OR (randomly[Title/Abstract])) OR (trial[Title/Abstract])) OR (clinical[Title/Abstract])) OR (clinical trial[Title/Abstract]))
#7	#1 AND#2AND#5AND#6

### 2.3 Study selection and data extraction

The search results from each database were imported into EndNote (version X21). First, duplicate records across databases were removed. Second, preliminary exclusion of irrelevant studies was performed by reviewing titles and abstracts. Finally, full-text articles were assessed to further exclude studies unrelated to this research. The extracted data included: (1) general publication information (author names, publication year, country, sample size of included studies); (2) patient characteristics (age, sex, days since ischemic stroke onset, etc.); (3) specific details of interventions in both treatment and control groups (treatment duration, therapeutic methods); (4) outcome measure details (assessment metrics). The screening and data extraction were conducted independently by two researchers. Any discrepancies were resolved through consultation with a third-party reviewer.

### 2.4 Study quality assessment

Two researchers independently assessed the risk of bias in included RCTs using the ROB 2.0 tool. The evaluation covered five domains: (1) randomization process, (2) deviations from intended interventions, (3) missing outcome data, (4) outcome measurement, (5) selection of reported results. The two researchers then cross-verified their assessments. Any discrepancies were resolved through consultation with a third researcher to reach consensus on the study's inclusion.

### 2.5 Statistical method

#### 2.5.1 Pairwise meta-analysis

Meta-analysis was performed using Review Manager 5.3, with assessment of heterogeneity and sensitivity analysis. Continuous outcomes were analyzed using mean difference (MD) as the effect measure, with pooled effect sizes and their 95% confidence intervals (CI) reported. Heterogeneity was evaluated among studies: when *I*^2^ ≤ 50%, indicating insignificant heterogeneity, a fixed-effects model was applied; when *I*^2^ > 50%, indicating substantial heterogeneity, a random-effects model was employed.

#### 2.5.2 Network meta-analysis

Network diagrams were generated using Stata 14.0 to visualize treatment duration relationships, where node size represented patient numbers per intervention and line thickness indicated direct comparison study counts between interventions.

To compare the therapeutic effects of different acupuncture treatment courses, Bayesian network analysis was conducted using ADDIS 1.16.6 with Markov chain Monte Carlo (MCMC) methods. Parameter settings included: 4 chains for simulation 50,000 simulation iterations and 20,000 adaptation iterations to eliminate initial value effects. Model convergence was confirmed when the Potential Scale Reduction Factor (PSRF) stabilized at 1. Inconsistency was assessed using MCMC models with identical parameters. The consistency model was adopted when random effects standard deviations approximated those of the inconsistency model, indicating no significant inconsistency. Evidence networks and ranking plots were generated, with higher outcome index improvement value indicating better efficacy (Rank 1 = best, Rank *N* = worst).

### 2.6 Subgroup analysis

Considering that neural recovery capacity may vary significantly with the stage of stroke and the severity of upper limb motor impairment, this study further conducted subgroup analyses based on baseline characteristics. Participants were stratified according to the stage of stroke, defined as hyperacute/acute phase (within 1 week after onset), subacute phase (within 12 weeks after onset), and chronic phase (more than 12 weeks after onset) (Joy and Carmichael, 2021). They were also classified by severity of upper limb motor dysfunction using the FMA-UE, with mild impairment corresponding to FMA-UE scores of 43–66, moderate impairment to scores of 29–42, and severe impairment to scores of 0–28 (Woytowicz et al., 2017).

For each subgroup, a Bayesian network meta-analysis consistent with the overall analysis was performed to integrate direct and indirect evidence. For the primary outcome measure FMA-UE, the differences in optimal intervention duration across various subgroups were individually assessed, with results visualized through forest plots and cumulative ranking probability plots.

### 2.7 Sensitivity analysis

When *I*^2^ ≥ 50%, sensitivity analyses were conducted using the one-study-removed method to identify potential sources of heterogeneity.

### 2.8 Publication bias

We generated a funnel plot to assess publication bias.

### 2.9 Evidence quality assessment

This study employed the GRADE approach to evaluate the quality of evidence. Given that all included studies were randomized controlled trials, the evidence quality was primarily assessed based on five downgrading factors: risk of bias, inconsistency, indirectness, imprecision, and publication bias. The certainty of evidence was classified four levels: high, moderate, low or very low. The summary of findings tables was prepared as a summary of the certainty of evidence using GRADEpro V.3.6.1.

## 3 Results

### 3.1 Search results

**Figure 1:**
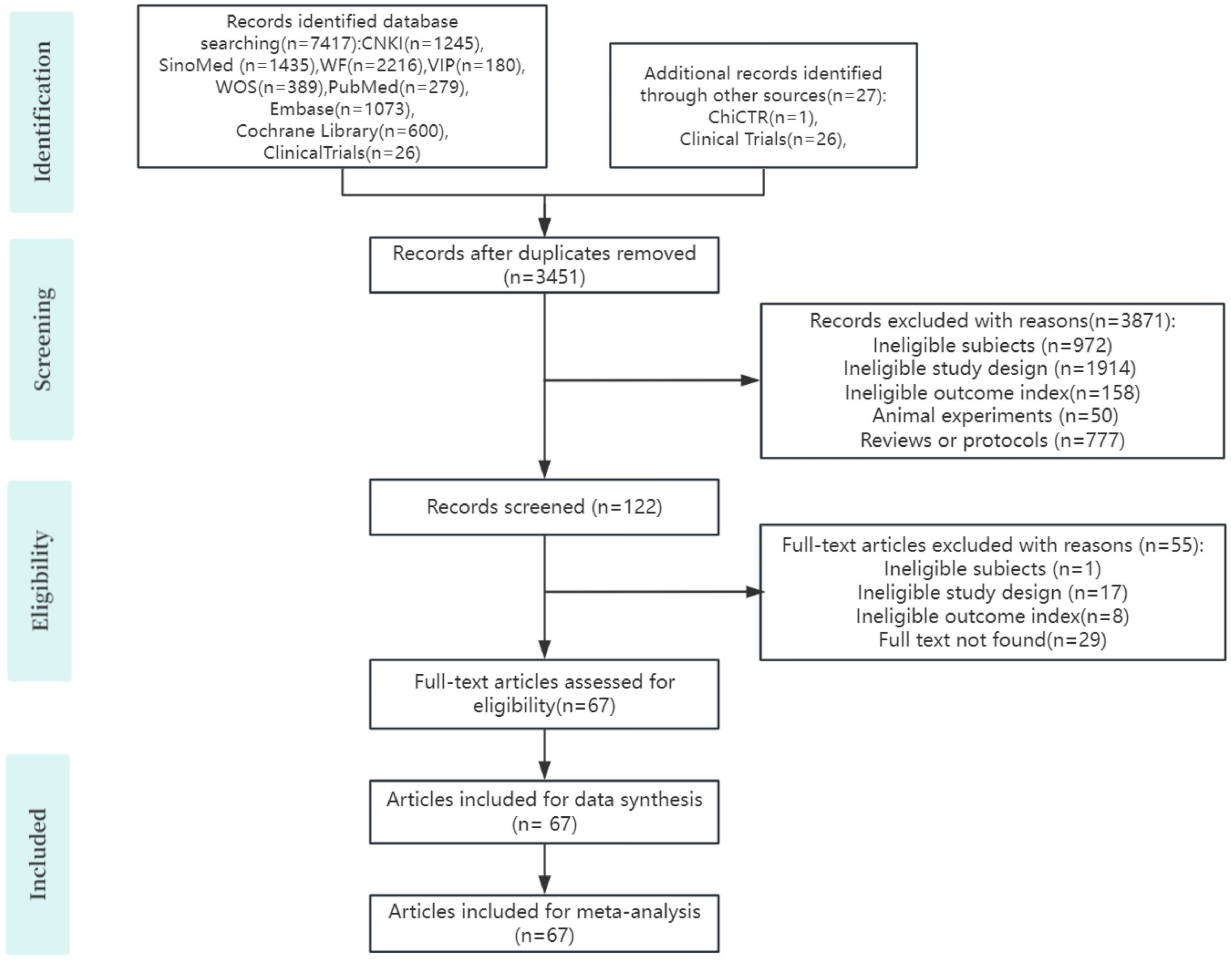
Selection of studies.

Following initial database searches, we identified 7,444 potentially relevant studies. After removing 3,451 duplicate records, 3,993 articles remained for title/abstract screening. This yielded 122 articles for full-text evaluation, from which 55 were excluded. Ultimately, 67 RCTs met inclusion criteria for this systematic review (Li L. Y. et al., 2021, 2022; Cui et al., 2021; Xu et al., 2015; Wang J. M. et al., 2021; Wang et al., 2023; Zhang et al., 2024, 2010; Xu, 2020; Yan, 2022; He et al., 2023; Hsieh et al., 2007; Gong and Xie, 2022; Bi et al., 2022; Wei et al., 2023; Liu et al., 2005; Lv et al., 2003; Ji, 2023; Xu et al., 2019; Yan et al., 2017; Wu et al., 2022; Yang et al., 2007; Liu, 2024; Bai et al., 2013; Tang et al., 2016; Yang, 2019; Zhong, 2021; Hou and Xu, 2022; Chen and Guan, 2022; Yu and Tian, 2023; Lang et al., 2013; Sun and Tu, 2024; Cao, 2022; Huang and Ou, 2021; Sun, 2020; Yin, 2021; Chen et al., 2014; Ding, 2024; Shen and Zou, 2025; Han and Shi, 2023; Wang, 2021; Pan and Jin, 2021; Xian et al., 2022; Ban et al., 2019; Kong, 2023; Xu, 2017; Ye et al., 2019; Xiong et al., 2020; Song, 2019; Zhang and Wang, 2019; Sang et al., 2023; Guo and Liu, 2022; Wang and Gong, 2022; Hou and Gao, 2024; Wang, 2022, 2024; Lu et al., 2025; Sha and Ma, 2020; Wu and Zhang, 2016; Yan et al., 2024; Qian, 2023; Liu et al., 2019; Li C. Y. et al., 2022; Zhou et al., 2023; Wang F. Q. et al., 2021; Fan et al., 2023; Li and Yuan, 2024). The selection flowchart is presented in Figure 1.

### 3.2 Characteristics of included studies

The analysis ultimately incorporated 67 studies, comprising 63 RCTs published in Chinese and 4 in English. All studies were published between 2003 and 2025, involving 5,635 participants. Sample sizes ranged from 20 to 200 participants, with most trials employing 1:1 allocation ratio. Patient ages spanned 22–85 years (except one RCT without specified age range). The included studies investigated various interventions comprising manual acupuncture, electroacupuncture, warm acupuncture, sham acupuncture, and their combinations with conventional therapy. The evaluated treatment courses spanned 2, 3, 4, 6, 8, 9, and 12 weeks. For outcome assessment, the Fugl-Meyer Assessment Upper Extremity (FMA-UE) scale was employed in 67 studies, the National Institutes of Health Stroke Scale (NIHSS) in 15 studies, and the Modified Barthel Index (MBI) in 12 studies. Complete details and results of all included studies are presented in Supplementary file S2.

### 3.3 Study quality assessment

**Figure 2:**
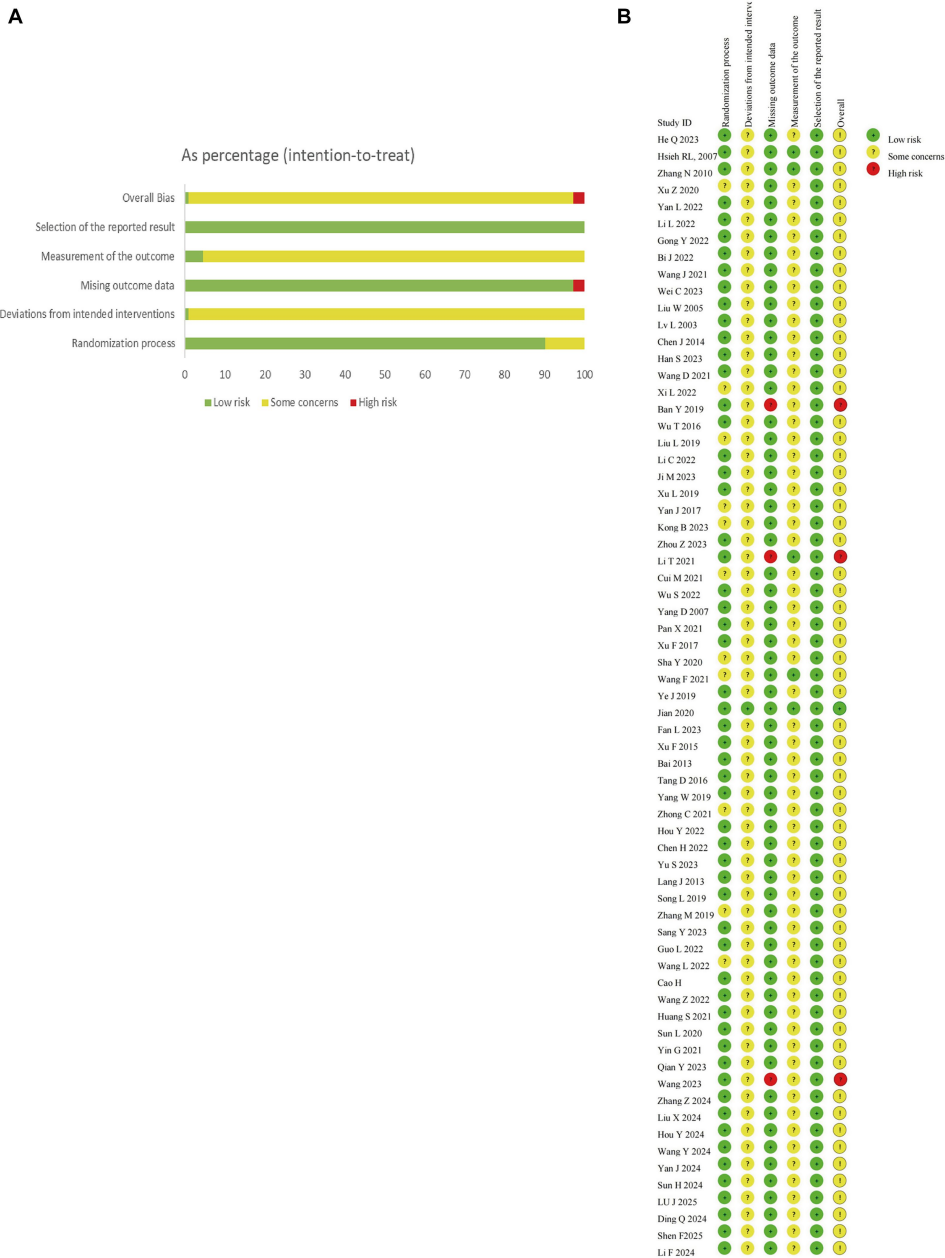
Results of quality assessment of included studies; **(A)** risk of bias graph; **(B)** risk of bias summary.

Among the 67 included studies, 11 studies (Cui et al., 2021; Xu, 2020; Yan et al., 2017; Zhong, 2021; Xian et al., 2022; Kong, 2023; Zhang and Wang, 2019; Wang and Gong, 2022; Sha and Ma, 2020; Liu et al., 2019; Wang F. Q. et al., 2021) only mentioned “randomization” without specifying the method and were therefore judged to have some risk of bias. The remaining studies described specific randomization methods such as random number tables or lottery and were rated as low risk of bias. Only one study (Xiong et al., 2020) implemented double-blinding, while the others did not report blinding of researchers. Considering the feasibility of blinding in acupuncture interventions, these studies were assessed as having some risk of bias due to deviations from intended interventions. Three studies (Li et al., 2021; Wang et al., 2023; Ban et al., 2019) had cases of participant dropout and were thus rated as high risk of bias, while five studies (Li et al., 2021; Zhang et al., 2010; Hsieh et al., 2007; Xiong et al., 2020; Wang et al. F. Q., 2021) used third-party outcome assessors and were judged as low risk of bias in outcome measurement. One study (Xiong et al., 2020) was rated as low risk of bias across all domains, three studies (Li et al., 2021; Wang et al., 2023; Ban et al., 2019) were assessed as high risk overall, and the remaining studies were judged to have some concerns of bias overall. The risk of bias assessments for included studies are presented in Figure 2, the detailed assessment results are shown in Supplementary file S3.

### 3.4 Pairwise meta-analysis

#### 3.4.1 FMA-UE

67 studies reported FMA-UE scores for acupuncture treatment of PIS-ULMD, including 2,829 patients in intervention groups and 2,806 in control groups. Heterogeneity testing revealed substantial between-study variability (*I*^2^ = 96%, *p* < 0.0001), warranting application of a random-effects model. The pooled analysis demonstrated statistically superior improvement in FMA-UE scores for acupuncture groups compared to conventional therapy alone (MD = 6.95, 95% CI: 5.89–8.00).

Given the substantial heterogeneity among included studies, we conducted subgroup analyses stratified by seven intervention courses: 2-week course, 3-week course, 4-week course, 6-week course, 8-week course, 9-week course and 12-week course subgroups. The results demonstrated no statistically significant difference between the 6-week course subgroup and control group (*p* > 0.05), while all other subgroups showed superior efficacy compared to controls (*p* < 0.05). See Supplementary file S4.

#### 3.4.2 NIHSS

15 studies involving 613 patients in the acupuncture group and 612 controls reported NIHSS scores for PIS-ULMD. Heterogeneity testing revealed significant heterogeneity among studies (*I*^2^ = 93%, *p* < 0.0001), warranting a random-effects meta-analysis. The analysis demonstrated that acupuncture significantly outperformed conventional therapy in improving neurological deficits, as evidenced by greater NIHSS score reduction (MD = 3.38, 95% CI: 2.35–4.40).

Due to the high heterogeneity among the included studies, further subgroup analysis was conducted based on intervention duration, categorizing studies into five subgroups: 4-week course, 6-week course, 8-week course, 9-week course, and 12-week course subgroups. The results showed that all subgroups exhibited superior efficacy compared to the control group (*p* < 0.05), as illustrated in Supplementary file S4.

#### 3.4.3 MBI

12 studies involving 514 patients in the acupuncture group and 516 controls reported Modified Barthel Index (MBI) scores for PIS-ULMD. Heterogeneity assessment revealed substantial heterogeneity among studies (*I*^2^ = 91%, *p* < 0.0001), necessitating a random-effects model for meta-analysis. The results demonstrated superior MBI score improvement in the acupuncture group compared to conventional treatment (MD = 8.33, 95% CI: 5.72–10.93), indicating statistically significant enhancement of upper limb motor function in PIS-ULMD patients.

Considering the high degree of heterogeneity (*I*^2^ = 91%) among studies, we conducted subgroup analyses by intervention duration (4-week course, 8-week course, 9-week course, and 12-week course subgroups). All subgroups showed significantly better therapeutic effects than the control group (*p* < 0.05). See Supplementary file S4.

### 3.5 Network meta-analysis

#### 3.5.1 FMA-UE

**Figure 3:**
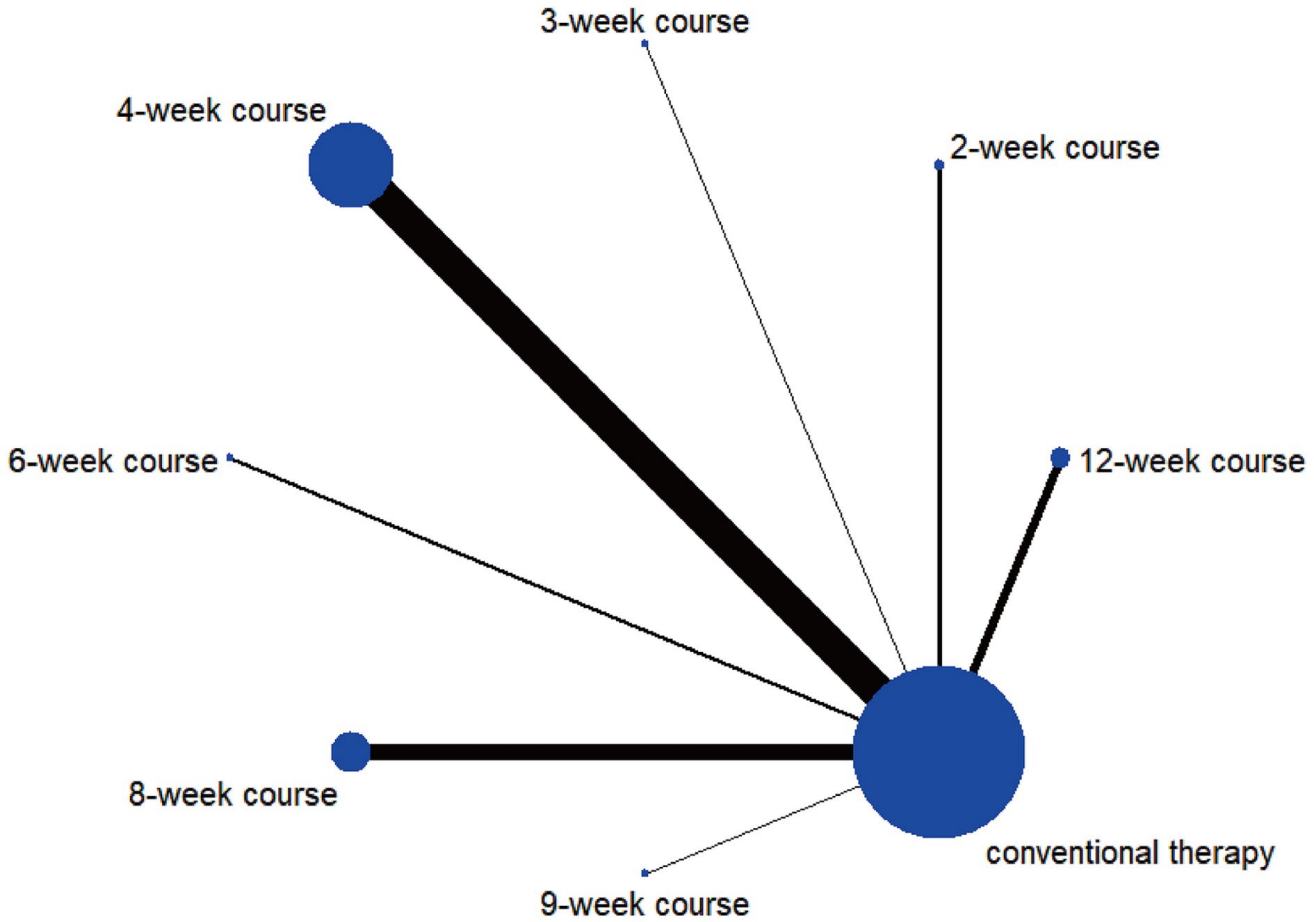
Network plot of FMA-UE.

The network evidence diagram results demonstrated that the included studies comprised seven intervention courses. In the network plot, nodes represent specific treatment courses, while connecting lines indicate direct comparisons between courses, with line thickness proportional to the number of comparative studies. Among current RCTs investigating acupuncture for PIS-ULMD, the frequency distribution of treatment courses was: 4-week course > 8-week course > 12-week course > 2-week course > 6-week course > 3-week course = 9-week course. See Figure 3.

**Figure 4:**
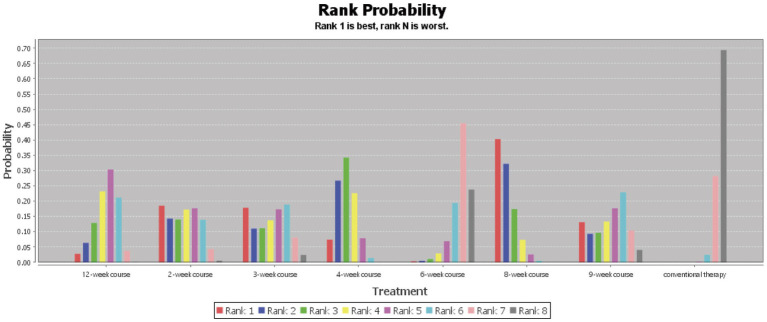
Ranking probability figure for FMA-UE.

**Figure 5:**
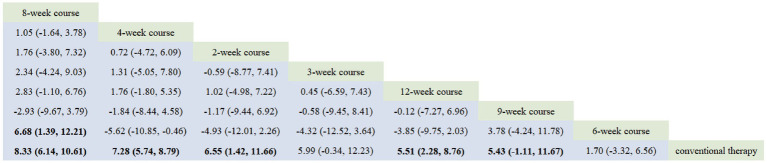
Network meta-analysis results for FMA-UE.

According to the PSRF results (values close to 1), see in Supplementary file S7. Both the consistency and inconsistency models were employed for network meta-analysis, along with Monte Carlo simulation iterations. Since the random-effects standard deviation and inconsistency standard deviation were approximately equal, we employed the consistency model for network meta-analysis and generated rank probability plots (Figure 4). As shown in Figures 4, 5, 8-week acupuncture course, 4-week acupuncture course, and 2-week acupuncture course ranked the top three in this study and were significantly more effective than conventional treatment. Among the seven acupuncture intervention courses investigated, 8-week acupuncture course was recommended as the most effective intervention period for improving FMA-UE scores.

#### 3.5.2 NIHSS

**Figure 6:**
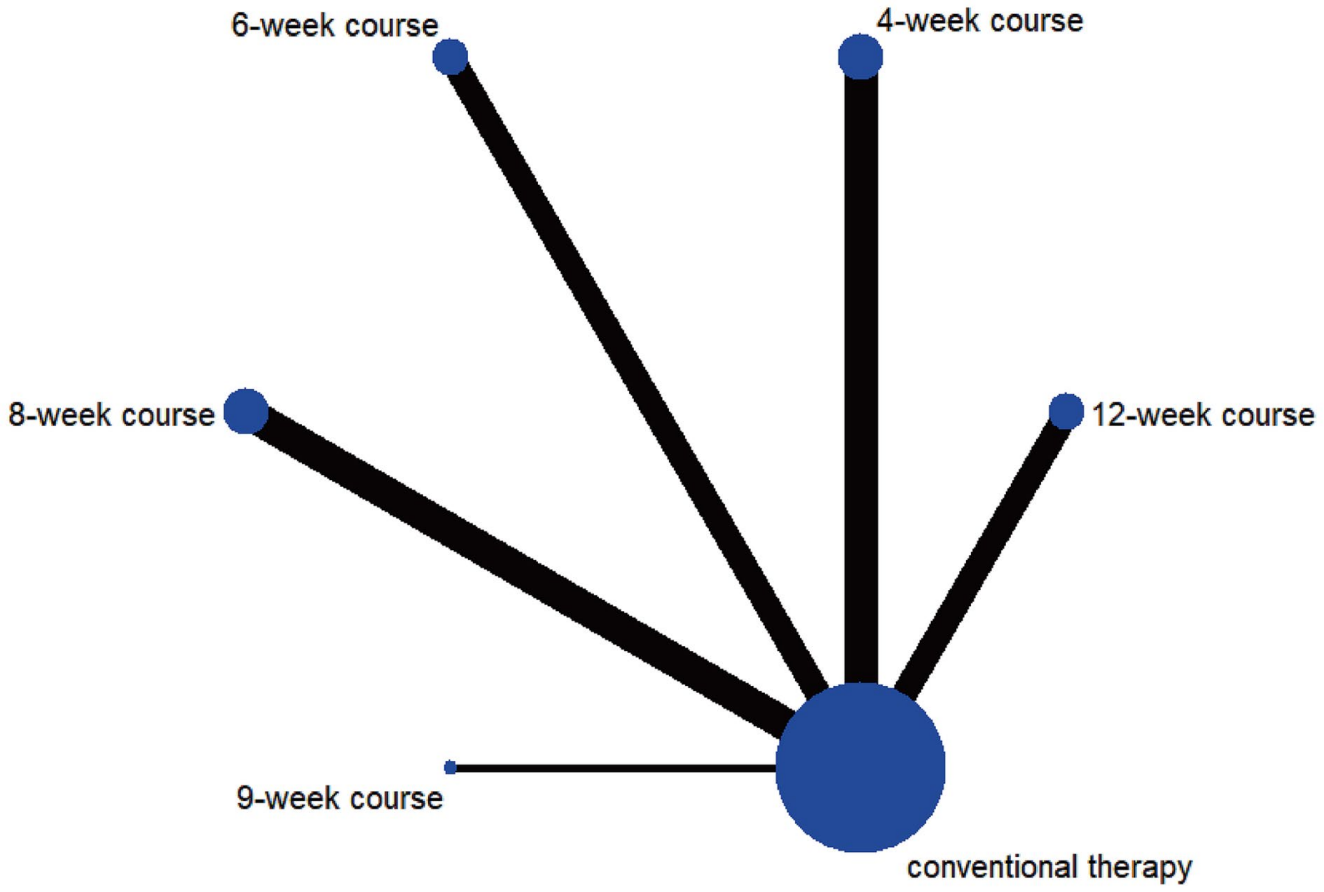
Network plot of NIHSS.

The network evidence map revealed the following frequency distribution of acupuncture intervention courses in current RCTs for PIS-ULMD treatment: 4-week course = 8-week course > 6-week course = 12-week course > 9-week course (Figure 6).

**Figure 7:**
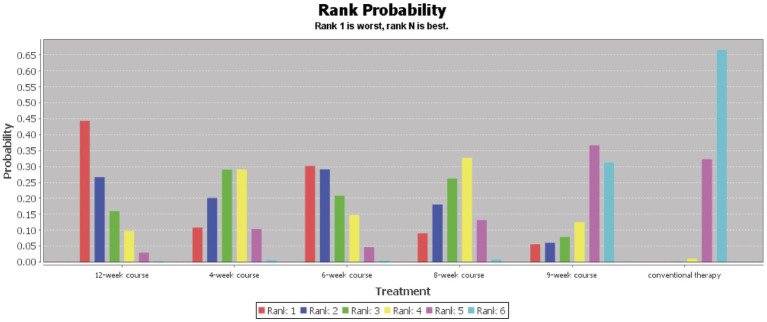
Ranking probability figure for NIHSS.

**Figure 8:**
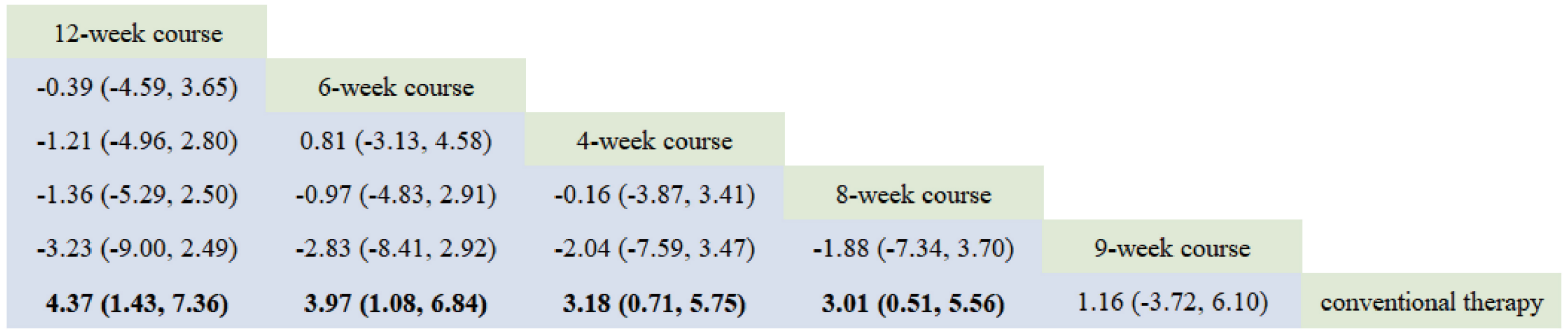
Network meta-analysis results for NIHSS.

Based on the PSRF results (values approximating 1), see in Supplementary file S7. Both consistency and inconsistency models were employed for network meta-analysis with Monte Carlo simulation iterations. Given the approximate equivalence between random-effects standard deviation and inconsistency standard deviation, the consistency model was selected for final network meta-analysis, producing rank probability plots (Figure 7). As demonstrated in Figures 7, 8, 12-week acupuncture course, 6-week acupuncture course, and 4-week acupuncture course ranked as the top three interventions in this study, all demonstrating significantly greater efficacy than conventional treatment. Among the five investigated acupuncture intervention courses, 12-week acupuncture course was identified as the most effective regimen for improving NIHSS scores.

#### 3.5.3 MBI

**Figure 9:**
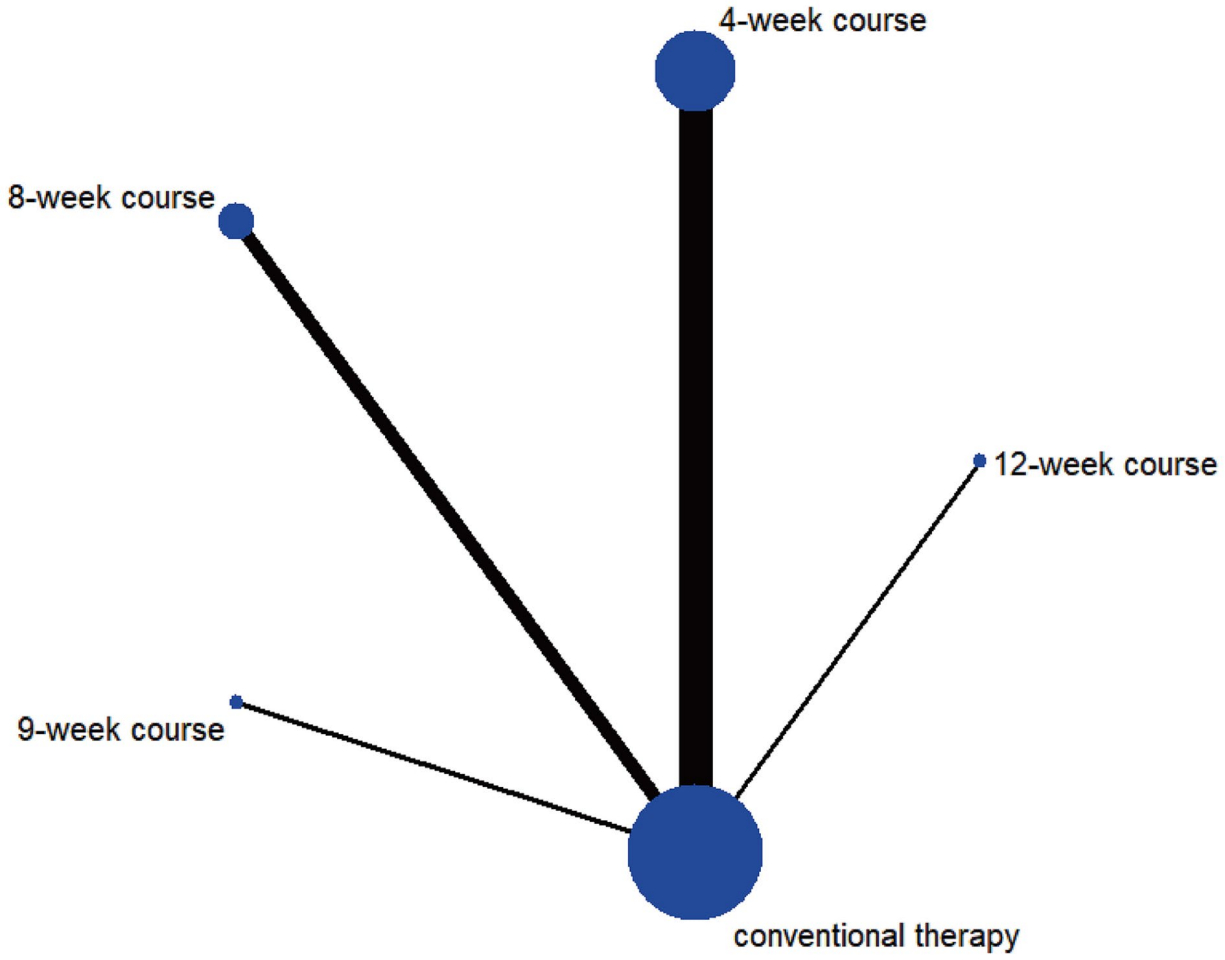
Network plot of MBI.

Network meta-analysis evidence ranking demonstrated the following frequency distribution of acupuncture treatment courses in current RCTs for PIS-ULMD: 4-week course > 8-week course > 9-week course = 12-week course (Figure 9).

**Figure 10:**
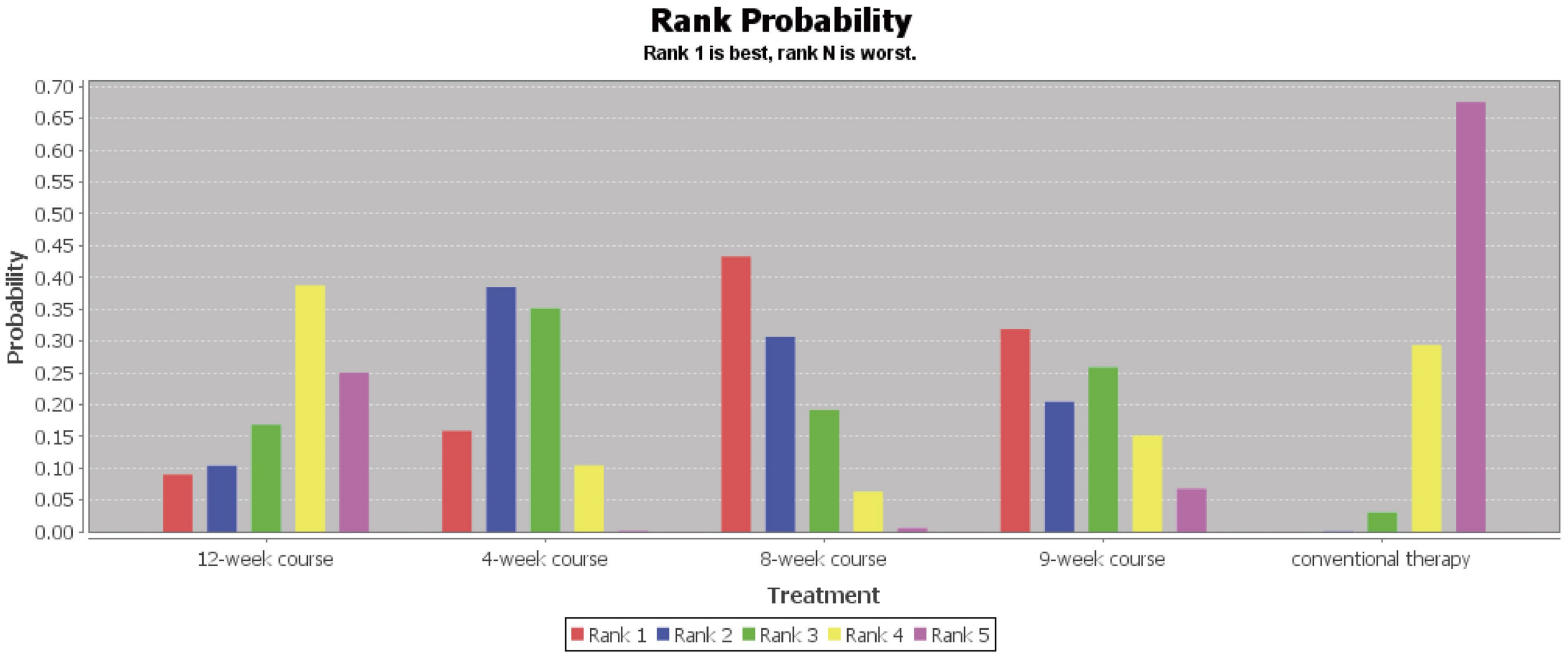
Ranking probability figure for MBI.

**Figure 11:**
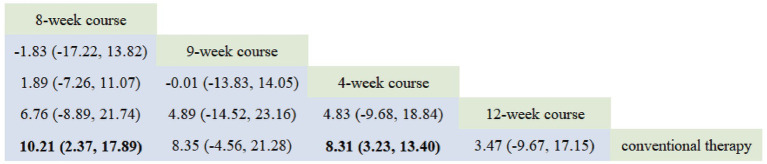
Network meta-analysis results for MBI.

Based on the PSRF results (values approximating 1), see in Supplementary file S7. Both consistency and inconsistency models were employed for network meta-analysis (NMA) with Monte Carlo simulation iterations. The random-effects standard deviation was found to be approximately equal to the inconsistency standard deviation. We therefore selected the consistency model for the final network meta-analysis and generated rank probability plots (Figure 10). As shown in Figures 10, 11, 8-week course, 4-week course, and 9-week course acupuncture interventions ranked as the top three treatments in this study. Among the four acupuncture treatment courses evaluated, 8-week acupuncture course was identified as the most effective regimen for improving MBI scores.

### 3.6 Sensitivity analysis

Sensitivity analysis was conducted in this study by sequentially excluding individual studies from each high-heterogeneity subgroup.

In FMA-UE, the pooled MD and *I*^2^ for the 4 W and 8 W groups remained within a relatively stable range after sequential exclusion of individual studies, indicating robust results. After excluding the study by Wang (2021) from the 6-week course group, the pooled MD was 5.21 (95% CI: 4.34–6.07, *p* < 0.001), and the *I*^2^ value decreased from 98 to 0%; after excluding the study by Yan et al. (2024) in the 12-week course group, the pooled MD was 5.38 (95% CI: 4.27–6.48, *p* < 0.001) with *I*^2^ declining from 87 to 16%, with both groups showing significantly reduced heterogeneity and maintaining statistically significant differences compared to controls. In NIHSS, after excluding the study by Gong and Xie (2022) in the 4-week course group, the pooled MD was 2.05 (95% CI: 1.35–2.75, *p* < 0.001) with *I*^2^ decreasing from 82 to 44%; after excluding Pan and Jin (2021) in the 6-week course group, MD was 2.42 (95% CI: 1.73–3.11, *p* < 0.001) with *I*^2^ dropping from 96 to 0%; after excluding Sang et al. (2023) in the 8-week course group, MD was 4.50 (95% CI: 3.49–5.50, *p* < 0.001) with *I*^2^ reducing from 94 to 1%; and after excluding Li C. Y. et al. (2022) in the 12-week course group, MD was 5.33 (95% CI: 4.20–6.46, *p* < 0.001) with *I*^2^ declining from 86 to 0%, with all groups maintaining statistically significant differences compared to control. In MBI, after excluding the study by Huang and Ou (2021) in the 4-week course group, the pooled mean difference was 7.24 (95% CI: 3.36–11.13, *p* < 0.001), with *I*^2^ decreasing from 92 to 83%, indicating reduced heterogeneity and a statistically significant difference compared to the control group (see Supplementary file S5).

### 3.7 Subgroup analysis

#### 3.7.1 Stage of stroke

We conducted a stratified analysis of 61 studies that reported clear duration of patient stroke course (6 studies did not explicitly specify the stroke course of included patients). The results demonstrated that, across patients in the hyperacute/acute phase, subacute phase, and chronic phase, the acupuncture group showed statistically significant greater improvements in FMA-UE scores compared to the conventional treatment alone. NMA using consistency models was performed to generate ranking probability plots, revealing significant differences in the optimal intervention durations for improving FMA-UE scores among different stroke phases. An 8-week acupuncture course regimen was optimal for the subacute phase (see Supplementary file S6).

#### 3.7.2 Severity of upper limb impairment

A stratified analysis was conducted on 66 studies that reported the baseline FMA-UE scores of included patients (one study did not clearly specify baseline FMA-UE scores). The results indicated that, compared to conventional treatment alone, acupuncture significantly improved FMA-UE scores in patients with PIS-ULMD, regardless of mild-to-moderate or severe impairment, with statistically significant differences. NMA using a consistency model was performed for each severity subgroup, and ranking probability plots were generated. The optimal duration of acupuncture intervention for improving FMA-UE scores differed significantly depending on the severity of upper limb motor impairment. For those with severe impairment, an 8-week course acupuncture intervention was recommended as the most effective duration (see Supplementary file S6).

### 3.8 Publication bias

Publication bias assessment was conducted for the outcome measures (FMA-UE, NIHSS, and MBI) across the 67 included studies. The funnel plot for FMA-UE demonstrated good symmetry, with most studies clustered in the upper portion, indicating minimal publication bias; however, some data points located at the bottom and outside of the funnel suggested possible small-study effects and potential heterogeneity. The funnel plots for NIHSS and MBI demonstrated poorer symmetry, suggesting potential publication bias. As shown in Supplementary file S8.

### 3.9 Evidence quality assessment

The evidence quality for different acupuncture courses in treating PIS-ULMD was as follows: low for upper limb motor function improvement (FMA-UE), very low for neurological impairment (NIHSS), and very low for quality of life (MBI), with the overall evidence quality ranging from low to very low. This was primarily due to limitations, inconsistency, and publication bias (detailed GRADE assessments are provided in Table 2 and Supplementary file S9).

**Table 2:** GRADE evidence profile for acupuncture treatment courses in PIS-ULMD.

Outcomes measures	Number of studies	Effective dose	Evidence quality assessment factors					Quality of evidence
	Patients	(95% CI)	Risk of bias	Inconsistency	Indirectness	Imprecision	Publication bias	
FMA-UE	67 (5635)	MD 6.95 [5.89,8.00]	−1 ①	−1 ②	0	0	0	Low
NIHSS	15 (1225)	MD 3.38 [2.35,4.40]	−1 ①	−1 ②	0	0	−1 ③	Very low
MBI	12 (1030)	MD 8.33 [5.72,10.93]	−1 ①	-1 ②	0	0	−1 ③	Very low

① Downgraded one level due to risk of bias because most of participants are from studies at moderate risk of bias. ② Downgraded one level due to substantial heterogeneity (*I*^2^ statistic value >50%). ③ Downgraded one level due to publication bias because the funnel plot indicates the existence of bias.

### 3.10 Adverse events

In this study, five publications explicitly documented the monitoring of adverse events (AEs) associated with acupuncture (He et al., 2023; Shen and Zou, 2025; Xu, 2017; Wu and Zhang, 2016; Yan et al., 2024). Among these, three studies reported no occurrence of AEs, while two randomized controlled trials (RCTs) recorded a total of eight AEs in the acupuncture treatment groups. One trial reported two cases of gastrointestinal reactions, two cases of abnormal liver function, and two cases of palpitation (He et al., 2023). Another trial documented two cases of infection at the acupuncture site (Wu and Zhang, 2016). The remaining studies did not explicitly mention safety outcomes or monitoring.

## 4 Discussion

### 4.1 Summary of the results

This study included 67 RCTs involving 5,635 PIS-ULMD patients across 7 different acupuncture courses, employing network meta-analysis to compare the effects of various intervention periods on upper limb motor function recovery using three outcome measures: FMA-UE (reported in 67 studies), NIHSS (15 studies), and MBI (12 studies).

Our findings demonstrate that acupuncture significantly improves upper limb motor function in PIS-ULMD patients compared to conventional therapy, with varying clinical efficacy across different treatment courses. For the primary outcome FMA-UE is the most widely used clinical measure for post-stroke upper limb motor recovery (Li et al., 2025), the optimal improvements occurred with 8-week course, 4-week course, and 2-week course. Secondary outcomes showed: NIHSS improvements were best with 12-week course, 6-week course, and 4-week course, while MBI improvements peaked with 8-week course, 4-week course, and 9-week course regimens. The observed discrepancy between NIHSS and FMA-UE results may stem from greater baseline NIHSS variability and milder neurological impairment in 8-week course groups, resulting in smaller measurable improvements. The collective evidence suggests 8-week acupuncture course may represent the optimal duration for simultaneously enhancing upper limb function and quality of life in PIS-ULMD patients.

### 4.2 Sources of heterogeneity

In FMA-UE, heterogeneity significantly decreased in both the 6-week course and 12-week course groups after excluding Wang (2021) and Yan et al.’s (2024) studies, respectively. This primarily stemmed from notable discrepancies in baseline characteristics between patients in these two studies and others within their respective groups. Additionally, Yan et al. (2024) employed a special “hand and foot acupuncture with twelve needles “acupuncture protocol, potentially amplifying intervention effect differences. In NIHSS, high heterogeneity in 4-week course, 8-week course, and 12-week course groups was also linked to inconsistent baseline neurological deficit severity, particularly significant baseline differences between patients in Gong and Xie (2022), Sang et al. (2023), and Li C. Y. et al.’s (2022) studies versus others in their groups. In the 6-week course group, Pan and Jin (2021) included patients with longer disease duration, who were in the chronic phase and had poorer neurological recovery potential, leading to greater divergence in outcomes from other studies in the same group. For MBI, high heterogeneity in the 4-week course group similarly stemmed from Huang and Ou (2021) study, where patients were also in the chronic phase, and significant differences in disease duration from other patients in the same group contributed to this variability.

High heterogeneity in this study may primarily stem from variations in patient baseline characteristics (e.g., severity of motor function impairment, disease stage) and intervention protocols. These findings suggest that results for 8 weeks, as one of the optimal intervention periods, may represent an average of heterogeneous responses, necessitating further validation through stratified analysis to yield more precise evidence. Consequently, to address the substantial heterogeneity observed in this study, we conducted additional subgroup analyses based on the phase after stroke and the severity of upper limb motor impairment. The results indicated that an 8-week acupuncture course intervention yielded the optimal outcomes for patients in the subacute phase and severe PIS-ULMD. We found that the optimal acupuncture treatment course varies depending on the patient’s baseline characteristics. An 8-week course of acupuncture is particularly effective for subacute, severe PIS-ULMD patients.

### 4.3 Comparison with similar studies

Ischemic stroke is a disease with high incidence, disability, and mortality rates in clinical practice, often causing varying degrees of limb dysfunction, where PIS-ULMD is particularly challenging to rehabilitate and significantly impacts patients’ quality of life. As a traditional and effective therapeutic approach, acupuncture can significantly improve functional impairments and enhance daily living abilities in PIS-ULMD patients. Our findings align with conventional meta-analyses by Feng et al. (2022), and Li H. P. et al. (2022), confirming acupuncture’s efficacy in post-stroke upper limb functional recovery. Huang et al. (2024) analyzed four treatment duration intervals (10-day, 2-week course, 3-week course, and 4-week course) for acute ischemic stroke, suggesting 4 weeks as potentially the most favorable duration based on NIHSS scores; however, the lack of comparisons beyond 4 weeks prevented determination of whether longer courses provide additional benefits or the optimal treatment period. This study is the first to elucidate the relationship between multiple treatment courses and clinical efficacy through network meta-analysis. Furthermore, existing meta-analyses on acupuncture for PIS-ULMD have primarily focused on different acupuncture methods (Zhang et al., 2024) and intervention timing (Zhuo et al., 2021). Currently, there remains a paucity of high-quality RCTs directly comparing the efficacy of different acupuncture courses for PIS-ULMD, indicating that standardized acupuncture protocols for PIS-ULMD still require further development.

### 4.4 Implications for clinical practice and future research

Acupuncture therapy exhibits cumulative effects, with therapeutic effects accumulating as the duration and frequency of acupuncture increase. However, upon reaching a certain level, the therapeutic effects reach a plateau or even decline (Cheng et al., 2022). Evidence for a precise efficacy plateau in acupuncture for PIS-ULMD is currently lacking. However, a related study on post-stroke wrist-hand functional reconstruction showed that electroacupuncture benefits change dynamically over time, featuring distinct phases of improvement, plateau, and decline (Wang et al., 2019). In line with the findings of this study, although the 12-week course involved a higher total intervention dose, its efficacy on the primary outcome measure was not significantly superior to that of the 8-week course. This observation aligns with the previously mentioned possibility of “acupuncture tolerance” in the introduction. We believe that the results of this study provide preliminary clinical evidence supporting the hypothesis that “acupuncture efficacy reaches a plateau.” However, due to the limited number of studies involving longer treatment durations included in this meta-analysis, the current evidence remains insufficient. Therefore, more high-quality randomized controlled trials are needed in the future to validate this hypothesis. Our results mark initial progress in exploring the optimal treatment duration of acupuncture for patients with PIS-ULMD, addressing a previous evidence gap. Despite the low quality of the current evidence, the findings may serve as a preliminary reference for selecting treatment cycles in clinical practice. Furthermore, this study revealed that the optimal treatment courses differ for motor function and overall neurological deficits, suggesting that future research should consider comprehensive selection of treatment courses based on patients’ motor abilities and neurological impairments.

### 4.5 Strengths and limitations

This study possesses several notable strengths. In contrast to previous research, it is the first to specifically investigate the effects of different acupuncture intervention courses on patients with PIS-ULMD, conducting subgroup analyses that address a critical gap in the literature regarding optimal acupuncture treatment duration for PIS-ULMD. Furthermore, the application of network meta-analysis (NMA) has significantly strengthened the robustness of the evidence by synthesizing both direct and indirect comparative data. However, this study has several limitations that should be acknowledged. Firstly, since our research primarily focused on acupuncture treatment duration, and sensitivity analysis revealed that specific acupuncture protocols had minimal impact on the overall study findings. This approach is also consistent with existing research on acupuncture dosage, which has similarly not emphasized technical variations (Wang et al., 2024). We consequently pooled these interventions for analysis. However, we acknowledge that variations in specific techniques remain a key limitation and potential source of heterogeneity. Secondly, Studies on acupuncture treatment durations beyond the 4-week and 8-week courses remain relatively limited. This evidence gap may affect the reliability of treatment ranking results, particularly for less-studied treatment courses. Thirdly, since all included studies were conducted in China, the generalizability of our conclusions is limited. Caution should be exercised when extrapolating these findings to other populations or clinical settings. Fourthly, safety analysis indicated that most studies did not record acupuncture-related adverse events. Especially since safety data for the 12-week treatment group were nearly absent (only one study reported such information). As a result, this meta-analysis cannot reliably assess whether extending treatment beyond 8 weeks increases the risk of adverse effects. Furthermore, factors such as lack of allocation concealment and unclear blinding methods in some studies may reduce the reliability of the conclusions in this review.

## 5 Conclusion

Acupuncture can effectively improve upper limb motor function, neurological function, and daily living activities in PIS-ULMD patients. For enhancing upper limb motor function, an 8-week acupuncture regimen may be more appropriate, particularly for patients in the subacute phase and severe PIS-ULMD. Future high-quality, large-sample, multicenter randomized controlled trials are warranted to further determine the optimal treatment course for acupuncture in managing PIS-ULMD.

## Data Availability

The original contributions presented in the study are included in the article/Supplementary material, further inquiries can be directed to the corresponding authors.

## References

[ref1] Bai Y. L. Li L. Hu Y. S. Wu Y. Xie P. J. Wang S. W. . (2013). Prospective, randomized controlled trial of physiotherapy and acupuncture on motor function and daily activities in patients with ischemic stroke. J. Altern. Complement. Med. 19, 684–689. doi: 10.1089/acm.2012.0578, PMID: 23600965

[ref2] Ban Y. C. Lin X. X. Geng X. F. Lu X. S. (2019). Clinical efficacy of acupuncture combined with modern rehabilitation technique on the upper extremity motor function recovery at early stage of stroke and analysis of the magnetic resonance diffusion tensor imaging. Mod. Med. J. 47, 439–442. doi: 10.3969/j.issn.1671-7562.2019.04.016

[ref3] Bi J. F. Li K. Zhang X. (2022). The efficacy of acupuncture for opening aperture and regaining consciousness on acute cerebral infarction and its influence on cognitive motor function. Clin. J. Chin. Med. 14, 57–59. doi: 10.3969/j.issn.1674-7860.2022.21.019

[ref4] Cao H. (2022). Effects of Xingnao Kaiqiao acupuncture combined with rehabilitation therapy on limb function and neurological function in patients with qi deficiency and blood stasis type cerebral infarction during recovery period. Inner Mongolia J. Tradit. Chin. Med. 41, 112–113. doi: 10.16040/j.cnki.cn15-1101.2022.02.019

[ref5] Chavez L. M. Huang S. S. Mac Donald I. Lin J. G. Lee Y. C. Chen Y. H. (2017). Mechanisms of acupuncture therapy in ischemic stroke rehabilitation: a literature review of basic studies. Int. J. Mol. Sci. 18:2270. doi: 10.3390/ijms18112270, PMID: 29143805 PMC5713240

[ref6] Chen J. Chen H. Zhong C. J. (2014). Analysis of therapeutic effect of acupuncture plus exercise therapy for treatment of 58 cases of patients with hemiplegia. Lab. Med. Clin. 92, 2087–2088. doi: 10.3969/j.issn.1672-9455.2014.15.018

[ref7] Chen H. L. Guan F. (2022). Clinical effect of acupuncture combined with rehabilitation therapy in the treatment of shoulder-hand syndrome after ischemic stroke. Chin. Med. Herald 19, 126–142. doi: 10.20047/j.issn1673-7210.2022.02.027

[ref8] Cheng Y. T. Sang P. Zhou H. (2022). Based on stroke to explore the influence of time factors on acupuncture efficacy. Shanghai J. Acupunct. Moxibust. 41, 617–621. doi: 10.13460/j.issn.1005-0957.2021.13.0050

[ref9] Chinese Society of Neurology, Chinese Stroke Society (2024). Chinese guidelines for diagnosis and treatment of acute ischemic stroke 2023. Chin. J. Neurol. 57, 523–559. doi: 10.3760/cma.j.cn113694-20240410-00221

[ref10] Cui M. J. Jiang L. M. Huang S. L. Xu Z. L. Zhang J. (2021). Analysis of the effect of early acupuncture and moxibustion on hemiplegia after acute cerebral infarction. Shenzhen J. Integr. Tradit. Chin. West. Med. 31, 49–52. doi: 10.16458/j.cnki.1007-0893.2021.21.020

[ref11] Ding Q. M. (2024). Observation on the application effect of acupuncture combined with functional training in limb dysfunction after cerebral infarction. Med. Forum 28, 149–152. doi: 10.19435/j.1672-1721.2024.30.042

[ref12] Fan L. W. Cai G. Y. Zhu H. M. Zhang R. P. (2023). Clinical study on scalp acupuncture combined with Bobath rehabilitation therapy for hemiplegia after cerebral infarction. New Chin. Med. 55, 166–170. doi: 10.13457/j.cnki.jncm.2023.12.034

[ref13] Feng Z. T. Lin R. Luo J. Xu M. Z. Tang C. Z. Cui S. Y. (2022). Meta-analysis of acupuncture combined with rehabilitation training in the treatment of upper limb motor dysfunction after ischemic stroke. J. Guangzhou Univ. Tradit. Chin. Med. 39, 703–711. doi: 10.13359/j.cnki.gzxbtcm.2022.03.040

[ref14] GBD 2021 Stroke Risk Factor Collaborators (2024). Global, regional, and national burden of stroke and its risk factors, 1990-2021: a systematic analysis for the global burden of disease study 2021. Lancet Neurol. 23, 973–1003. doi: 10.1016/S1474-4422(24)00369-7, PMID: 39304265 PMC12254192

[ref15] Gong Y. T. Xie B. (2022). Clinical value analysis of early acupuncture treatment on hemiplegia rehabilitation after acute cerebral infarction. Inner Mongol. J. Tradit. Chin. Med. 41, 106–107. doi: 10.16040/j.cnki.cn15-1101.2022.08.039

[ref16] Guo L. L. Liu J. (2022). Effect of acupuncture in the treatment of patients with post-stroke upper limb hemiplegia and its influence on limb motor function. Chin. Med. Herald 19, 140–3, 52. doi: 10.20047/j.issn1673-7210.2022.04.028

[ref17] Han S. X. Shi N. (2023). Clinical effect analysis of acupuncture combined with rehabilitation training for upper limb spasticity after cerebral infarction. J. Chin. Foreign Med. Pharm. Res. 2, 78–80. doi: 10.3969/j.issn.2096-6229.2023.01.026

[ref18] He Q. Fan L. Zhu J. Lian T. T. (2023). Effects of warm acupuncture combined with mirror therapy on functional recovery, homocysteine, and brain-derived neurotrophic factor in elderly patients with hemiplegia after cerebral infarction. Hainan Med. J. 34, 929–932. doi: 10.3969/j.issn.1003-6350.2023.07.004

[ref19] Hou Y. X. Gao Y. Q. (2024). Effect of hand and foot twelve needle assisted comprehensive training on walking ability and limb function in hemiplegic patients after cerebral infarction. Reflexol. Rehabil. Med. 5, 22–4,8. Available at: https://d.wanfangdata.com.cn/periodical/ChlQZXJpb2RpY2FsQ0hJTmV3UzIwMjQwNzA0EhNmc2hsZnlrZnl4MjAyNDA1MDA3Gghnb2ZjdXBsNA%3D%3D

[ref20] Hou Y. J. Xu S. C. (2022). Clinical effect of acupuncture and moxibustion in the treatment of neck and shoulder pain of hemiplegia after stroke. Clin. Res. Pract. 7, 147–149. doi: 10.19347/j.cnki.2096-1413.202207042

[ref21] Hsieh R. L. Wang L. Y. Lee W. C. (2007). Additional therapeutic effects of electroacupuncture in conjunction with conventional rehabilitation for patients with first-ever ischaemic stroke. J. Rehabil. Med. 39, 205–211. doi: 10.2340/16501977-0032, PMID: 17468788

[ref22] Huang S. P. Ou R. Q. (2021). Clinical effect of electroacupuncture combined with modified constraint-induced movement therapy on limb function recovery in stroke patients with hemiplegia. Inner Mongolia J. Tradit. Chin. Med. 40, 108–109. doi: 10.16040/j.cnki.cn15-1101.2021.08.060

[ref23] Huang S. Wang Y. Wu Y. Huang P. Dong Y. Zhang Q. . (2024). Acupuncture for acute ischemic stroke: a systematic review and meta-analysis of randomized controlled trials. Integr. Med. Res. 13:101092. doi: 10.1016/j.imr.2024.101092, PMID: 39686968 PMC11646781

[ref24] Ji M. X. (2023). Application effect of acupuncture rehabilitation nursing in upper limb paralysis after cerebral infarction. Chin. J. Urban Rural Enterp. Hyg. 38, 213–215. doi: 10.16286/j.1003-5052.2023.10.080

[ref25] Joy M. T. Carmichael S. T. (2021). Encouraging an excitable brain state: mechanisms of brain repair in stroke. Nat. Rev. Neurosci. 22, 38–53. doi: 10.1038/s41583-020-00396-7, PMID: 33184469 PMC10625167

[ref26] Koh C. L. Pan S. L. Jeng J. S. Chen B. B. Wang Y. H. Hsueh I. P. . (2015). Predicting recovery of voluntary upper extremity movement in subacute stroke patients with severe upper extremity paresis. PLoS One 10:e0126857. doi: 10.1371/journal.pone.0126857, PMID: 25973919 PMC4431803

[ref27] Kong B. F. (2023). Clinical observation of harmonizing yin-yang meridian acupuncture for post-ischemic stroke hemiplegia. Guangming J. Chin. Med. 38, 3807–3810. doi: 10.3969/j.issn.1003-8914.2023.19.037

[ref28] Kwakkel G. Kollen B. Twisk J. (2006). Impact of time on improvement of outcome after stroke. Stroke 37, 2348–2353. doi: 10.1161/01.STR.0000238594.91938.1e, PMID: 16931787

[ref29] Lang J. Y. Zhuang L. X. He J. Jia C. Zhou Z. H. Ke L. P. (2013). Randomized controlled study on Jin’s three needle therapy on spastic hemiplegia after ischemic stroke. Shanghai J Acupunct Moxibust. 32, 440–443. doi: 10.3969/j.issn.1005-0957.2013.06.440

[ref30] Li C. Y. Chen H. Luo G. Q. Wu X. N. Liu L. Jiang C. . (2022). An functional MRI study of effects of acupuncture combined with rehabilitation training on recovery of upper limb motor function in ischemic stroke patients. Neural Inj. Funct. Reconstr. 17, 76–8,88. doi: 10.16780/j.cnki.sjssgncj.20201044

[ref31] Li D. Li R. Song Y. Qin W. Sun G. Liu Y. . (2025). Effects of brain-computer interface based training on post-stroke upper-limb rehabilitation: a meta-analysis. J. Neuroeng. Rehabil. 22:44. doi: 10.1186/s12984-025-01588-x, PMID: 40033447 PMC11874405

[ref32] Li L. Y. Qiao J. Ding H. Y. (2022). Effects of Tongdu Tiaoshen acupuncture in treatment of patients with acute cerebral infarction. Med. J. Chin. People's Health. 34, 111–113. doi: 10.3969/j.issn.1672-0369.2022.11.035

[ref33] Li Y. Wang Q. Liu X. L. Hui R. Zhang Y. P. (2023). Effect of the physical rehabilitation program based on self-care ability in patients with acute ischemic stroke: a quasi-experimental study. Front. Neurol. 14:1181651. doi: 10.3389/fneur.2023.1181651, PMID: 37360351 PMC10288520

[ref34] Li T. Wang S. Y. Song Y. W. Yang Z. Zhang S. Wang S. . (2021). Clinic research of acupuncture full cover methods in treating hemiplegic upper-extremity dysfunction secondary to brain infarction. Clin. J. Tradit. Chin. Med. 33, 952–957. doi: 10.16448/j.cjtcm.2021.0538

[ref35] Li F. X. Yuan L. Z. (2024). Clinical effect of acupuncture on cerebral infarction hemiplegia. Pract. Clin. J. Integr. Tradit. Chin. West. Med. 24, 100–120. doi: 10.13638/j.issn.1671-4040.2024.13.028

[ref36] Li H. P. Zhai Y. B. Xing J. Wang J. L. (2022). Acupuncture and moxibustion in the treatment of post-stroke patients with upper limb spastic hemiplegia: meta-analysis. World Chin. Med. 17, 196–214. doi: 10.3969/j.issn.1673-7202.2022.02.010

[ref37] Liu X. F. (2024). Therapeutic effects of scalp acupuncture combined with mirror therapy on upper limb motor function and activities of daily living in hemiplegic patients after cerebral infarction. Chin. Health Care 42, 28–31. Available at: https://d.wanfangdata.com.cn/periodical/ChlQZXJpb2RpY2FsQ0hJTmV3UzIwMjQwNzA0EhpRS0JKQkQyMDI0MjAyNDAxMTkwMDAwODE1NRoIZ29mY3VwbDQ%3D

[ref38] Liu L. Chen S. Q. Wei J. Xu X. B. Jing X. H. Wang L. P. (2019). Effect of Wang’s “hand-foot twelve needles” acupuncture on neuroplasticity of primary motor cortex in ischemic stroke patients. Global Tradit. Chin. Med. 12, 385–389. doi: 10.3969/j.issn.1674-1749.2019.03.015

[ref39] Liu W. P. Lin Z. Z. Tan H. Y. Cai C. L. Xing Z. H. (2005). The effect of acupuncture treatment on the early rehabilitation in the patients with acute cerebral stroke. J. Hebei North Univ. 22, 37–38. doi: 10.3969/j.issn.2095-1396.2005.02.016

[ref40] Lu J. H. Yin X. C. Li Y. J. (2025). Effects of scalp acupuncture and body acupuncture combined with occupational therapy on cognition, muscular spasticity, and fine motor of affected limbs in patients with spastic hemiplegia of upper limbs after stroke. New Chin. Med. 57, 83–87. doi: 10.13457/j.cnki.jncm.2025.04.017

[ref41] Lv L. J. Shen L. Y. Fan G. Q. Zhu L. P. (2003). Clinical study on treatment of acupuncture on cerebral infarction with upper extremity motor disfunction. Zhejiang J. Integr. Tradit. Chin. West. Med. 13, 14–16. doi: 10.3969/j.issn.1005-4561.2003.01.005

[ref42] Nakayama H. Jørgensen H. S. Raaschou H. O. Olsen T. S. (1994). Recovery of upper extremity function in stroke patients: the Copenhagen stroke study. Arch. Phys. Med. Rehabil. 75, 394–398. doi: 10.1016/0003-9993(94)90161-9, PMID: 8172497

[ref43] Pan X. X. Jin Z. (2021). Analysis of therapeutic effect of Tongli Shuji acupuncture on patients with stroke. China Contin. Med. Educ. 13, 163–167. doi: 10.3969/j.issn.1674-9308.2021.33.044

[ref44] Pluta R. Januszewski S. Czuczwar S. J. (2021). The role of gut microbiota in an ischemic stroke. Int. J. Mol. Sci. 22:915. doi: 10.3390/ijms22020915, PMID: 33477609 PMC7831313

[ref45] Qian Y. (2023). Effects of acupuncture and moxibustion combined with rehabilitation training on limb motor function and nerve function in patients with cerebral infarction and hemiplegia. China Foreign Med. Treatment 42, 1–4, 9. doi: 10.16662/j.cnki.1674-0742.2023.29.001

[ref46] Rodgers H. Bosomworth H. Krebs H. I. van Wijck F. Howel D. Wilson N. . (2019). Robot assisted training for the upper limb after stroke (RATULS): a multicentre randomised controlled trial. Lancet 394, 51–62. doi: 10.1016/S0140-6736(19)31055-4, PMID: 31128926 PMC6620612

[ref47] Sang Y. Chen Y. F. Hu B. (2023). Clinical study on Yin-Yang balance penetration combined with rehabilitation training for upper limb spasticity after stroke. New Chin. Med. 55, 168–171. doi: 10.13457/j.cnki.jncm.2023.08.034

[ref48] Sha Y. J. Ma Z. Y. (2020). Clinical efficacy observation of warm acupuncture in the treatment of hemiplegia during the recovery period of ischemic stroke. Med. Diet Health 18:72+4. Available at: https://d.wanfangdata.com.cn/periodical/CiFQZXJpb2RpY2FsQ0hJU29scjlTMjAyNTEwMjEwOTUwNDYSEHl4c2x5amsyMDIwMjIwNDUaCHp4MzJsZmJy

[ref49] Shen F. L. Zou T. (2025). Effect of acupuncture therapy combined with motor rehabilitation training on limb motor function in patients with acute stroke hemiplegia. J. Huaihai Med. 43, 57–60. doi: 10.14126/j.cnki.1008-7044.2025.01.014

[ref50] Song L. J. (2019). A study on effect of acupuncture combined with rehabilitation training on disorder of limb function and neurological function in patients with cerebral infarction. New Chin. Med. 51, 219–221. doi: 10.13457/j.cnki.jncm.2019.07.065

[ref51] Sun L. W. (2020). Effect of acupuncture combined with early rehabilitation therapy on stroke patients. Med. J. Chin. People's Health. 32, 93–95. doi: 10.3969/j.issn.1672-0369.2020.23.035

[ref52] Sun H. A. Tu Q. (2024). Xingnao kaiqiao acupuncture combined with comprehensive rehabilitation training for stage I shoulder-hand syndrome after ischemic stroke: a clinical observation. J. Pract. Tradit. Chin. Med. 40, 355–358. Available at: https://d.wanfangdata.com.cn/periodical/CiFQZXJpb2RpY2FsQ0hJU29scjlTMjAyNTEwMjEwOTUwNDYSEHN5enl5enoyMDI0MDIwNzMaCDFscHJ5dmJu

[ref53] Tang D. Wu W. P. Sun X. H. (2016). Tongjing acupuncture combined with functional training in the treatment of post-stroke shoulder-hand syndrome: a randomized controlled trial. J. Clin. Acupunct. Moxibust. 321, 26–29. doi: 10.19917/j.cnki.1005-0779.2016.01.009

[ref54] Wade D. T. Langton-Hewer R. Wood V. A. Skilbeck C. E. Ismail H. M. (1983). The hemiplegic arm after stroke: measurement and recovery. J. Neurol. Neurosurg. Psychiatry 46, 521–524. doi: 10.1136/jnnp.46.6.521, PMID: 6875585 PMC1027442

[ref55] Wang D. W. (2021). Study on the effect of traditional Chinese medicine acupuncture and early rehabilitation training on neurological function and limb motor function of patients with hemiplegia after stroke. Reflexol. Rehabil. Med. 2, 13–15. Available at: https://d.wanfangdata.com.cn/periodical/ChlQZXJpb2RpY2FsQ0hJTmV3UzIwMjQwNzA0EhNmc2hsZnlrZnl4MjAyMTA3MDA1Gghnb2ZjdXBsNA%3D%3D

[ref56] Wang Z. D. (2022). Effects of acupuncture combined with dynamic-static balance rehabilitation training on muscle spasticity, limb motor function, and activities of daily living in post-stroke hemiplegic patients. Reflexol. Rehabil. Med. 3, 24–27. Available at: https://d.wanfangdata.com.cn/periodical/ChlQZXJpb2RpY2FsQ0hJTmV3UzIwMjQwNzA0EhNmc2hsZnlrZnl4MjAyMjE5MDA3Gghnb2ZjdXBsNA%3D%3D

[ref57] Wang Y. C. (2024). Therapeutic effect analysis of acupuncture combined with hemiplegic limb rehabilitation training on post-cerebral infarction hemiplegia. Chin. Health Care 42, 68–70. Available at: https://d.wanfangdata.com.cn/periodical/ChlQZXJpb2RpY2FsQ0hJTmV3UzIwMjQwNzA0EhpRS0JKQkQyMDI0MjAyNDAxMTkwMDAwODE2OBoINnV3cWVoZWE%3D

[ref58] Wang D. Y. Dong X. Wang B. (2019). Electroacupuncture for post-stroke wrist and hand functional reconstruction: a dose-response relationship study. J. Li-shizhen Tradit. Chin. Med. 30, 1914–1915. doi: 10.3969/j.issn.1008-0805.2019.08.041

[ref59] Wang L. Gong Z. R. (2022). Clinical observation on treating upper limb dysfunction in hemiplegic patients with ischemic stroke by dynamic scalp acupuncture plus task oriented mirror. Clin. J. Chin. Med. 14, 106–109. doi: 10.3969/j.issn.1674-7860.2022.28.027

[ref60] Wang Y. Lu M. Liu R. Wang L. Wang Y. Xu L. . (2023). Acupuncture alters brain's dynamic functional network connectivity in stroke patients with motor dysfunction: a randomised controlled neuroimaging trial. Neural Plast. 2023, 1–14. doi: 10.1155/2023/8510213, PMID: 37383656 PMC10299883

[ref61] Wang J. M. Wang Z. Y. Wang D. (2021). Study on penetrating acupuncture at affected-side acupoints along yin meridians for acute cerebral infarction with hemiplegia. Chin. J. Convalesc. Med. 30, 715–717. doi: 10.13517/j.cnki.ccm.2021.07.016

[ref62] Wang X. Xiao L. Xiao L. Tian C. Liu Y. Dai X. (2024). The dose–effect relationship of acupuncture on limb dysfunction after acute stroke: a systematic review and meta-analysis. Front. Neurol. 15:1341560. doi: 10.3389/fneur.2024.1341560, PMID: 38481941 PMC10933065

[ref63] Wang F. Q. Zhong J. G. Lai Q. J. Luo D. Y. Luo Q. Jiang N. . (2021). Randomized controlled study on Jin’s three needle therapy on spastic hemiplesia after ischemic stroke. World J. Integr. Tradit. West. Med. 16, 1284–1289. doi: 10.13935/j.cnki.sjzx.210722

[ref64] Wei C. Y. Ma Y. B. Li X. H. (2023). Therapeutic efficacy of Tongdu Tiaoshen acupuncture in acute cerebral infarction patients with hemiplegia. Lingnan J. Emerg. Med. 28, 53–55. doi: 10.3969/j.issn.1671-301X.2023.01.018

[ref65] Woytowicz E. J. Rietschel J. C. Goodman R. N. Conroy S. S. Sorkin J. D. Whitall J. . (2017). Determining levels of upper extremity movement impairment by applying a cluster analysis to the Fugl-Meyer assessment of the upper extremity in chronic stroke. Arch. Phys. Med. Rehabil. 98, 456–462. doi: 10.1016/j.apmr.2016.06.023, PMID: 27519928 PMC5299057

[ref66] Wu S. F. Shi J. F. Zhong X. P. Wu A. W. (2022). Clinical study on acupuncture combined with rehabilitation exercise for shoulder-hand syndrome after stroke. New Chin. Med. 54, 190–193. doi: 10.13457/j.cnki.jncm.2022.05.044

[ref67] Wu T. Zhang X. (2016). Study on acupuncture "regulating meridians and viscera" method combined with rehabilitation training for promoting motor function recovery in early-stage post-stroke hemiplegic patients. J. Emerg. Trad. Chin. Med. 25, 1241–1244. doi: 10.3969/j.issn.1004-745X.2016.06.102

[ref68] Xian L. X. Li R. M. Zhu H. F. Pan G. J. Xiao S. L. Wu G. R. (2022). Clinical observation on Xingnao Kaiqiao acupuncture therapy in the treatment of motor dysfunction in early cerebral infarction. J. Guangzhou Univ. Tradit. Chin. Med. 39, 831–836. doi: 10.13359/j.cnki.gzxbtcm.2022.04.017

[ref69] Xiong J. Zhang Z. Ma Y. Li Z. Zhou F. Qiao N. . (2020). The effect of combined scalp acupuncture and cognitive training in patients with stroke on cognitive and motor functions. Neuro Rehabil. 46, 75–82. doi: 10.3233/NRE-192942, PMID: 32039871

[ref70] Xu F. (2017). Randomized parallel controlled study of acupuncture and Moxibustion combined with ipsilateral limb forced exercise in the treatment of upper limb dysfunction after ischemic stroke. J. Pract. Tradit. Chin. Intern. Med. 31, 79–81. doi: 10.13729/j.issn.1671-7813.2017.10.29

[ref71] Xu Z. Y. (2020). Improvement effect of early acupuncture physiotherapy and nursing intervention on paralyzed limb function in patients with acute ischemic stroke. New Mom New Born:212. Available at: https://d.wanfangdata.com.cn/periodical/ChlQZXJpb2RpY2FsQ0hJTmV3UzIwMjQwNzA0Eg1teXNqMjAyMDI5MjA5Gghnb2ZjdXBsNA%3D%3D

[ref72] Xu F. Li H. L. Zhang Q. (2015). Acupuncture combined with rehabilitation training for treatment of shoulder-hand syndrome after ischemic stroke: a randomized controlled trial. Chin. J. Trauma Disabil. Med. 23, 141–142. doi: 10.13214/j.cnki.cjotadm.2015.16.107

[ref73] Xu L. L. Zhang Y. E. Cao J. P. (2019). Clinical study on Xiaoxingnao acupuncture combined with rehabilitation measures for hemiplegia in convalescence of ischemic stroke. New Chin. Med. 51, 241–243. doi: 10.13457/j.cnki.jncm.2019.08.072

[ref74] Yan L. Q. (2022). Intervention effect of acupuncture combined with rehabilitation training on motor dysfunction after stroke. J. Med. Inform. 35, 171–173. doi: 10.3969/j.issn.1006-1959.2022.01.043

[ref75] Yan J. Fang W. H. Guo Z. C. (2024). Study on the therapeutic effect of the "twelve needles for hands and feet" acupuncture technique in the treatment of spastic hemiplegia caused by cerebral infarction. Jilin J. Chin. Med. 44, 599–603. doi: 10.13463/j.cnki.jlzyy.2024.05.024

[ref76] Yan J. Jiang H. J. Yang Q. Tian Y. Gao H. Y. (2017). Post-stroke upper extremity function treated with acupuncture at Zhongzhu and Houxi. J. Shandong Second Med. Univ. 39, 188–190. doi: 10.16846/j.issn.1004-3101.2017.03.010

[ref77] Yang W. F. (2019). Acupuncture therapy combined with rehabilitation training for treatment of upper limb spasticity after cerebral infarction. World Latest Med. Inf., 180–181. doi: 10.19613/j.cnki.1671-3141.2019.80.089

[ref78] Yang D. F. Lin X. D. Yu Z. M. Wu Z. B. Wu X. F. Lin J. F. (2007). Effects of combined acupuncture with Bobath therapy on spasticity and motor function in post-stroke hemiplegia. Liaoning J. Tradit. Chin. Med. 34, 215–216. doi: 10.3969/j.issn.1000-1719.2007.02.066

[ref79] Yang A. Wu H. M. Tang J. L. Xu L. Yang M. Liu G. J. (2016). Acupuncture for stroke rehabilitation. Cochrane Database Syst. Rev. 2016:Cd004131. doi: 10.1002/14651858.CD004131.pub3, PMID: 27562656 PMC6464684

[ref80] Ye J. J. Dong Y. W. Lin X. F. Yang L. D. (2019). Clinical study on scalp acupuncture combined with balance function training for post-stroke balance dysfunction. New Chin. Med. 51, 232–235. doi: 10.13457/j.cnki.jncm.2019.07.069

[ref81] Yin G. D. (2021). Efficacy of warm-acupuncture on motor function in patients with spastic hemiplegia after stroke. Clin. J. Chin. Med. 13, 71–73. doi: 10.3969/j.issn.1674-7860.2021.17.022

[ref82] Yu S. Tian R. D. (2023). Effect of acupuncture and moxibustion combined with rehabilitation exercise therapy on limb motor function and hemorheology in patients with post stroke hemiplegia. Reflexol. Rehabilitat. Med. 4, 8–11. Available at: https://d.wanfangdata.com.cn/periodical/ChlQZXJpb2RpY2FsQ0hJTmV3UzIwMjQwNzA0EhNmc2hsZnlrZnl4MjAyMzE1MDAzGghnb2ZjdXBsNA%3D%3D

[ref83] Zhang N. X. Liu G. Z. Yao Q. H. Li W. J. Huang Y. Wang A. M. . (2010). Effects of warming-reinforcing acupuncture combined with rehabilitation training on the early motor function of hemiparalysis patients caused by ischemic brain stroke: a randomized and controlled study. Chin. Acupunct. Moxibust. 30, 441–445. doi: 10.13703/j.0255-2930.2010.06.007 20578377

[ref84] Zhang Z. J. Tao L. H. Ma X. Q. Zeng M. Zhu M. H. (2024). Clinical study on electroacupuncture combined with upper limb rehabilitation training for post-stroke stage I shoulder-hand syndrome. New Chin. Med. 56, 125–128. doi: 10.13457/j.cnki.jncm.2024.07.025

[ref85] Zhang M. Wang Y. P. (2019). Clinical observation on acupuncture combined with rehabilitation training in the treatment of ischemic stroke with upper limb spasm. Guangming J. Chin. Med. 34, 1236–1238. doi: 10.3969/j.issn.1003-8914.2019.08.040

[ref86] Zhang D. Zou W. Zhang B. Guo P. (2024). Scalp acupuncture for post-stroke spastic hemiparesis: a systematic review and meta-analysis. Medicine (Baltimore) 103:e37167. doi: 10.1097/MD.0000000000037167, PMID: 38428878 PMC10906645

[ref87] Zhong C. Y. (2021). Clinical effect of acupuncture combined with rehabilitation training on shoulder-hand syndrome after ischemic stroke. Reflexol. Rehabilitat. Med. 2, 34–40. Available at: https://d.wanfangdata.com.cn/periodical/ChlQZXJpb2RpY2FsQ0hJTmV3UzIwMjQwNzA0EhNmc2hsZnlrZnl4MjAyMTE3MDExGghnb2ZjdXBsNA%3D%3D

[ref88] Zhou Z. Q. Chao H. F. Huang H. P. (2023). Clinical observation of acupuncture combined with core stability rehabilitation training for post-stroke hemiplegia. J. Pract. Tradit. Chin. Med. 39, 2241–2243. Available at: https://kns.cnki.net/kcms2/article/abstract?v=BpvOe4q1DEciLBsLAtja06nRfCEo5RR_PLwF3dOthjoBaMjfNyeC_hnk-pllwH9qahfO-C8x44DrByfvrZ5gTkskcZEcVZQu8xLFBQ12-NQcQDB06lGCv_6czR66eBPkIcWi9XiAX3-eZJfjLgYwL_hPpZxfaeU43SSYvwKrurmyjtVE-AuevQuzySVWTKOnbcvjwiUWLGs=&uniplatform=NZKPT&language=CHS

[ref89] Zhu T. Zhou Y. Dai A. Li S. Zhou L. Zhang X. . (2024). Efficacy of acupuncture and rehabilitation therapy on brain function activation area and neurological function in ischemic stroke: a systematic review and meta-analysis. PLoS One 19:e0298547. doi: 10.1371/journal.pone.0298547, PMID: 38394111 PMC10889652

[ref90] Zhuo Y. Xu M. Deng S. Zhang Y. Lu X. Wu B. . (2021). Efficacy and safety of dissimilar acupuncture intervention time-points in treating stroke: a systematic review and network meta-analysis. Ann Palliat Med. 10, 10196–10212. doi: 10.21037/apm-21-1127, PMID: 34498479

